# Postgraduate Physiotherapy Training in a Quandary - Ramifications of Corona virus pandemic Lockdown: A Survey-based Study

**DOI:** 10.30476/jamp.2021.89189.1367

**Published:** 2021-07

**Authors:** SHAILESH GARDAS, SHRUTI NAIR, POTHIRAJ PITCHAI, VRUSHALI PANHALE

**Affiliations:** 1 Department of Neurophysiotherapy, Mahatma Gandhi Missions College of Physiotherapy, Navi Mumbai, India; 2 Department of Cardiovascular & Respiratory Physiotherapy, MGM College of Physiotherapy, Navi Mumbai, India; 3 Department of Community Physiotherapy, MGM College of Physiotherapy, Navi Mumbai, India; 4 Department of Musculoskeletal Physiotherapy, MGM College of Physiotherapy, Navi Mumbai, India

**Keywords:** COVID-19, Education, Pandemic, Survey

## Abstract

**Introduction:**

Coronavirus disease-2019 (COVID-19) has disrupted clinical services and postgraduate training across the world. Hence, this survey was conducted to understand the impact of pandemic on Physiotherapy post-graduate (PG) education.

**Methods:**

It was a cross-sectional, observational study. A total of 254 Physiotherapy PG trainees were recruited through snowball sampling from Physiotherapy colleges across the state of Maharashtra. A 34-item structured questionnaire was developed, based on available literature, to evaluate the impact of COVID-19 pandemic on four domains: academic training, clinical training, research activities, and concerns faced by a PG trainee. The face validity of this questionnaire was assessed by six academicians and their suggestions were examined. Subsequently, it was piloted on five PG trainees before administering it to the participants. The validated questionnaire was then circulated via various social media platforms and personal contacts using Google form. Descriptive statistics were summarized as frequencies/ percentages. McNemar’s test was used to determine the differences on a dichotomous dependent variable between the two related groups using SPSS software.

**Results:**

Overall 131 trainees provided complete responses. Although 85% (n=111) of them claimed they attended PG teaching activities through online mode, almost 67% (n=101) disagreed to have achieved their learning objectives. A vast majority (91%, n=119) of them felt that their specialty related practical training was severely affected, and 98% (n=129) reported that reduced caseload had impacted their clinical learning. Also, 70% (n=54) of final year PGs had difficulty in recruiting new participants for their dissertation. Spread of infections to family (98%), commuting in public transport (98%), uncertainty about exam dates (91%), and competency development in specialty areas (96%) were some of their concerns.

**Conclusion:**

COVID-19 pandemic had impacted various domains of Physiotherapy PG program such as academic, clinical and research areas. Regulatory authorities should take serious consideration and devise strategies to overcome it.

## Introduction

Corona virus disease 2019 (COVID-19), caused by severe acute respiratory syndrome coronavirus 2 (SARS-CoV-2), is a newly emergent virus first recognized in Wuhan, China, in December 2019. Rapid worldwide spread of COVID-19 prompted the World Health Organization (WHO) to declare it as ‘pandemic’ on 11^th^ of March 2020 ( 1, 2). In the wake of COVID-19 affecting India, Maharashtra was declared as the worst affected state, accounting for one third of all cases in the country ( 3).

Like most of the governments across the world, Indian government imposed a total lockdown from 25^th^ March 2020 along with re-enforcing social distancing and infection control guidelines to curb the pandemic ( 4). India had observed four phases of nationwide lockdown, which was extended up to 31 May 2020 ( 5). As a result, all regular health care services in the hospitals, nursing homes and clinics were closed and elective surgeries deferred, except for the emergency services ( 6). Universities and all other educational organizations across the world, including India, reacted quickly to this crisis by announcing immediate closure. Thus, from mid-March 2020 onwards UG and PG training of students from health sciences courses came to a halt. Social distancing, in response to the pandemic, led to the complete transition of academic teaching from classroom and clinical interactions to virtual sessions conducted online ( 7). Moreover, the assessment and evaluation of students were also done in online settings. All these could possibly cause an unprecedented impact on medical education as seen in previous studies in India ( 4, 8).

In recent times, researchers worldwide have studied the impact of COVID-19 on postgraduate education and training across varied specialties in medical residents from endoscopy, ophthalmology, orthopedics, to cardiothoracic specialty ( 6, 9- 12). Their findings reflected a profound blow on their education and training due to the closing of non-emergency hospitals, cancellation of elective surgeries and social distancing norms. Besides, the trainee’s psychological state has also been proven to be affected as observed by increased burnout, anxiety and stress reported in endoscopy trainees ( 9). A recent study done by Kapasia et al. (2020) in UG & PG learners of West Bengal, India, revealed major disruption of their academic activities due to COVID 19 lockdown ( 4). Upadhaya et al.’s study on the impact of COVID on orthopedic trainees in northern India has recommended measures to be taken by government and educational organizations to mitigate its long-term impact and ensure implementation of satisfactory mechanisms to overcome it ( 11).

Since physiotherapy (PT) is a direct contact practice, PG trainees require hands-on clinical practice and consistent patient interaction for prolonged duration to develop technical skills for effective patient care. PG program is directed towards rendering competency in specialty clinical skills to promote professional development. It encompasses various specialties like musculoskeletal PT, Neuro PT, Cardiopulmonary PT, Community PT, and Sports PT. Along with this, it consists of rigorous two years of research work inclusive of preparation of synopsis, recruitment of study participants, and undertaking of the study intervention protocols. This necessitates a steady inflow of patients/participants in the hospital set-up. Recent studies conducted on trainees of various medical specialties during the COVID era have shown a disruption in their training and education program ( 9- 14), but none has been reported on Physiotherapy students. 

Looking at the present scenario and the global footprint, COVID-19 does not seem to go away any time soon. Therefore, the present study was an attempt to assess the impact of the COVID-19 related pandemic on post-graduate teaching and learning.

## Methods

A cross-sectional, observational study was carried out using a 34-item self-reported questionnaire exclusively designed for Indian population. Its content element was drawn from previous studies conducted in different medical specialties across the world ( 9- 12). Overall, there were 254 Physiotherapy PG students recruited using snowball sampling from colleges affiliated to government university as well as deemed universities across the state of Maharashtra. The indigenous questionnaire was designed by the consensus of two authors having expertise in Physiotherapy education and training. It focused on the four domains of a PG training namely impact on academic training, clinical training, research activities, and concerns faced by a PG trainee. Responses were scored through a five-point Likert scale ranging from strongly agree to strongly disagree for most of the items, whereas few required dichotomous responses. The face validity of the questionnaire was assessed by two senior academicians with an academic experience of more than 14 years who were experts in the field of PG training. It was assessed in successive stages by requesting individual experts to evaluate the content relevance and simplicity of individual items and the entire set of items (questionnaire) as a tool followed by the iterative loops of consensus panel revisions. The consensus panel consisting of four academicians were requested to identify the lacuna in the questionnaire with regards to the location of the items, grammatical structure, and correct scaling. Few questions of the tool were then reframed, deleted, added, and modified considering their suggestions. Additional space was provided for them to give feedback and fill in deficient areas, if any.

The developed questionnaire was then piloted on five PG trainees belonging to different training levels to check for clarity and comprehension of the questions. Few items in the questionnaire were again modified based on their feedback, and the questionnaire was finalized to be used for the survey.

This cross-sectional online survey-based study was conducted from October 10, 2020 to October 15, 2020. The purpose of the study, the time required to complete it and permission to withdraw if deemed unsuitable was mentioned in the google form before the start of the survey questionnaire. A successful return of completed survey was considered as consent by the participant. The questionnaire was uploaded on Google form and the generated link was circulated amongst the participants. One representative from each institute was identified who served as the contact point. They were personally approached to explain the purpose of the study and discuss the workflow. These contact points (participants) were requested to forward the questionnaire links to their batchmates/peers through their common class e-mail ID and WhatsApp groups. Follow up and reminders to submit the filled questionnaires was ensured by personally contacting these representatives (contact points) after 24 hours. This way the authors aimed to target two hundred and fifty-four participants largely recruited using exponential non-discriminative snowball sampling technique. A second reminder was given to submit the completed questionnaire after a span of 48 hours. Submission of partially completed questionnaire was not accepted for analysis. The survey was closed on the 15^th^ of October at 10 pm. No financial or other incentives were provided for participation. The responses submitted were checked for Duplication, and then were pooled, analyzed and summarized.

### Statistical Analysis

SPSS (Statistical Package for the Social Sciences) version 21 (IBM Corp) was used for statistical analysis. All categorical variables were summarized as frequencies/ percentages. Participant’s age group was expressed as mean and standard deviation. For items with a five‐point Likert scale and a positive response set, the “strongly agree” and “agree” categories were combined, as were the “neutral,” “strongly disagree,” and “disagree” categories, so that responses fell into 1 of 2 categories: “agree” or “disagree.” Likewise, for items with a negative response set, the “neutral” category was combined with the “agree” and “strongly agree” categories. Response frequencies for the survey questions were thus determined and displayed in graphical formats. The McNemar’s test was used to determine if there were differences on a dichotomous dependent variable between the two related groups. Participant’s response before lockdown was considered as pre-test, and during lockdown as post-test. We considered p< 0.05 as statistically significant.

### Ethical Consideration

The study was commenced after approval from Institutional Review Board of MGM’s College of Physiotherapy, Maharashtra, India with ethical code; MGM/COP/IRRC/141/2020 dated 5th October 2020. The work was carried out in accordance with the Declaration of Helsinki alongside guaranteeing the anonymity of the participants.

## Results

### Participants’ Characteristics

In total, 149 respondents (out of 254) participated in this survey (response rate being 59%). After excluding incomplete (n=15) and duplicate (n=3) responses, 131 completed questionnaires were analyzed. Demographic details of the study participants are presented in Table 1.

**Table1:** The participant’s characteristics

Characteristics	Frequency in Percentage(n)
**Gender**	
Male	10(13)
Female	90(118)
Mean Age (S.D) in years	24.98(0.73)
**Admission year**	
2017	10(13)
2018	30(40)
2019	60(78)
**Batch**	
I MPT	41(54)
II MPT	59(77)
**PG Specialization**	
Musculoskeletal PT	40(52)
Community PT	21(27)
Cardiovascular & Respiratory PT	21(27)
Neuro PT	12(17)
Sports PT	5(7)
Pediatrics PT	1(1)
**University**	
MUHS	89(117)
Deemed	11(14)

SD: Standard Deviation, MPT: Master of Physiotherapy, PG: Post Graduation, PT: Physiotherapy.

Over 93% (n=122) of the participants belonged to institutions that were attached to a hospital dedicated to COVID-19. Among 131 participants, 68% (n=89) were reporting to college during lockdown. Out of those 89 participants, 68% (n=60) were attending college every day. However, only 28% (n=25, out of 89) of the participants were going to clinical postings daily, and the remaining 72% (n=64, out of 89) reported to their clinical postings less frequently in a week. 

On comparing the time spent in activities like attending clinical duties, Continuing Medical Education (CME), self-studying before and during the lockdown, it was observed that there was a significant deviation, as summarized in Table 2.

**Table2:** Frequency of the participant’s daily sessional activities before and during the lockdown

Activities	Before Lockdown Frequency in % (n)		During Lockdown Frequency in % (n)		p
	1-3 hours	4-6 hours	1-3 hours	4-6 hours	
Time spent in clinical duties	15 (20)	85 (111)	76 (100)	24 (31)	0.016*
Attending CME's/ Webinars	92 (120)	8 (11)	44 (57)	56 (74)	0.021*
Online surfing for academic/research purpose	79 (104)	21 (27)	45 (59)	55 (72)	0.052*
Self-study	76 (100)	24 (31)	56 (74)	44 (57)	0.011*
Recreational activities	82 (107)	18 (24)	62 (81)	38 (50)	0.029*
Social media	82 (107)	18 (24)	45 (59)	55 (72)	0.042*

* McNemar Test (p < 0.05 as significant), CME: Continuing Medical Education, n: denotes number of respondents.

### Impact of COVID-19 pandemic on PG Academic Training

The majority of the participants (86%, n=113) claimed that their PG teaching schedule was continued even after implementation of lockdown. Table 3 shows the duration of online classes and journal club sessions attended by participants before and during the lockdown. Although 85% (n=111) of the participants claimed to attend PG teaching activities via online mode, almost 67% (n=101) disagreed to have achieved their learning objectives.

**Table3:** Academic activities before and during COVID-19 lockdown

	Before Lockdown Frequency in % (n)	During Lockdown Frequency in % (n)	p
**Duration of Classes (Hours/week)**			
1-8 hours	88 (115)	85 (112)	0.031*
> 8 hours	12 (16)	15 (19)	
**Journal club (Sessions/ month)**			
1-3 sessions	79 (104)	59 (77)	0.014*
> 3 sessions	21 (27)	41 (54)	

* McNemar Test (p < 0.05 as significant) , n: denotes number of respondents.

**Figure 1:**
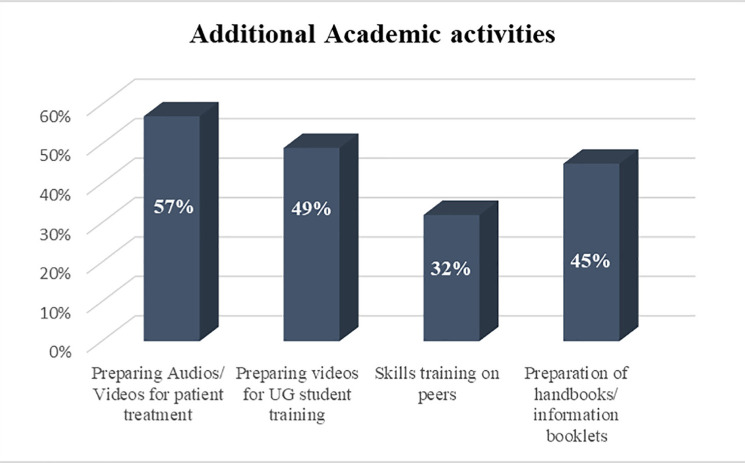
The participants’ multiple responses on additional academic activities they were engaged in during the lockdown

Apart from regular teaching activities through virtual medium, it was observed that participants were also involved in additional academic activities like preparing videos/audios, preparing information booklets, etc., as displayed in Figure 1. Even though they participated in the above activities, 91% (n=119) of them felt that their specialty related practical training was severely affected, and 84% (n=110) felt that they did not achieve the necessary hands-on skills due to the ongoing pandemic.

### Impact of COVID-19 pandemic on PG Clinical Training

Out of the total number of participants, 78% (n=102, out of 131) were deployed in all COVID- related clinical services. The majority of the participants agreed that their clinical exposure was affected due to the pandemic. Clinical exposure was reported to be affected as follows: 95% (n=125, out of 131) in outpatient (OPD) unit, 79% (n=102, out of 131) in inpatient department (IPD), and 57% (n=75, out of 131) in Intensive Care Unit (ICU), as reported by the participants. Table 4 shows the participants’ responses to the number of patients treated in non-COVID areas (clinical areas tending to patients not infected with COVID), before and during the lockdown. Nearly 63% (n=82) of the participants said that they did not get to work exclusively in their PG specialty area.

**Table4:** Participants’ responses to the patients treated in Non-COVID areas before and during the lockdown

	Before Lockdown		During Lockdown		p
	< 15 cases/ day	>15 cases/ day	< 15 cases/ day	>15 cases/ day	
OPD(n)	70	61	116	15	0.028*
IPD(n)	54	77	91	40	0.012*
ICU(n)	87	44	108	23	0.095

* McNemar Test (p < 0.05 as significant), n denotes the number of respondents.

**Figure 2:**
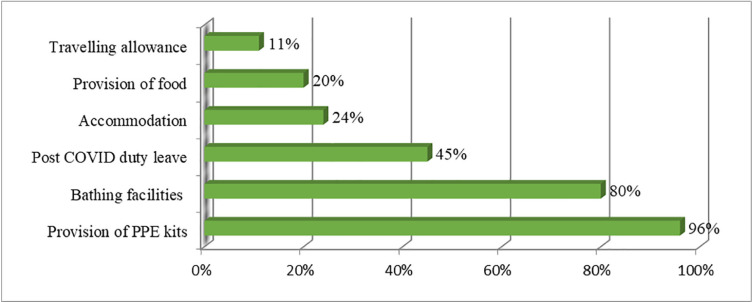
The facilities provided by institutions during COVID clinical duties

Based on their multiple responses, 93% (n=95) of the respondents were involved in COVID ICU services, 78% (n=80) in COVID wards, 29% (n=30) in post-COVID rehabilitation OPD, and 13% (n=13) were engaged in tele-rehabilitation. Almost 25% (n=26) of the participants involved in COVID services had three rotations per week, 55% (n=56) had one to two rotations, whereas only 20% (n=20) had to report daily for COVID duties. A significant proportion (89%, n=91) of the participants had received suitable personal protective equipment (PPE) training before commencing COVID duties. Figure 2 depicts the facilities provided by the institution during their COVID postings.

Almost 89% (n=117) of the participants reported that the pandemic had caused a change in their clinical service approach, so that their basic assessment skills (67%) and management skills (55%) were affected. Based on their multiple response, 95% (n=112) of them claimed they were involved in minimal contact practice, 38% (n=45) in tele-rehabilitation sessions, 31% (n=36) in no-contact practice, and 15% (n=18) were involved in practice through information booklets. 

A substantial percentage of participants reported that reduced patient flow in the hospital had affected their clinical learning pertaining to their specialty (98%, n=129); their peer learning experience was affected due to reduced contact and learning from the seniors (92%, n=120); they faced difficulty in presenting cases on patients in the current situation (90%, n=118). Only a small proportion of participants agreed that they were able to assess (23%) and treat (26%) patients thoroughly as compared to before COVID-19 pandemic. Furthermore, 57% (n=74) felt that COVID-19 pandemic had deterred their confidence to face the upcoming PG examination, whereas 26% (n=34) were unsure about it. Additionally, 38% (n=50) felt that COVID-19 pandemic had impacted their preparedness to be a good clinician, whereas an equal number of them (36%, n=47) expressed uncertainty in this regard.

### Impact of COVID-19 pandemic on research activity

Out of 77 final year PG students, before the lockdown, 35% (n= 27) were involved in data collection, 45% (n= 35) had completed data, and 19% (n=15) had submitted their dissertation. However, during the lockdown, most of them (70%, n=54 out of 77) had difficulty in recruiting new participants. Their multiple response on other research related difficulties is shown in Table 5. Keeping the ongoing pandemic in mind, about 57% (n=75) of the study participants claimed that university made certain exemptions/provisions to help them finish their dissertation. Apart from academic program, 15% (n=19) were also engaged in COVID-related research, either directly or indirectly.

**Table5:** Difficulties faced by the participants to conduct research activities

Reasons given by participants*	Frequency (%,n)
Reduced number of research participants from OPD/IPD/institutions/community	93% (n=85)
Unwillingness of participants to come for screening and follow up	91 %(n=83)
Interventional/assessment-based study designs	82% (n=75)
Restrictions on use of specialized equipment’s as per study protocol	79% (n=72)
Delay in response from the university regarding approval	79% (n=72)
Delay in meeting guide for discussion/signatures etc.	66% (n=60)

* Multiple responses possible

Table 6 summarizes the suggestions given by the participants in an event of continuation of lockdown during this pandemic. Additionally, the participants also suggested arrangement of simulation labs for practice, provision of prophylactic drugs, and allowances as specified by the central government for health-care workers as measures to support them.

**Table6:** The participants’ suggestions

Suggestions*	Frequency (%, n)
Modification in the rules for training and exit exams	98 % (n=129)
Extension of term for clinical training/data collection	76% (n=99)
Relaxation in sample size and duration of study intervention	92% (n= 120)
Provision for change in study design from intervention to survey based	83 % (n= 109)
Provision of more exclusive travel facilities for healthcare workers (including medical students)	96 % (n=126)

* Multiple responses possible

### Health and career-related concerns

**Figure 3:**
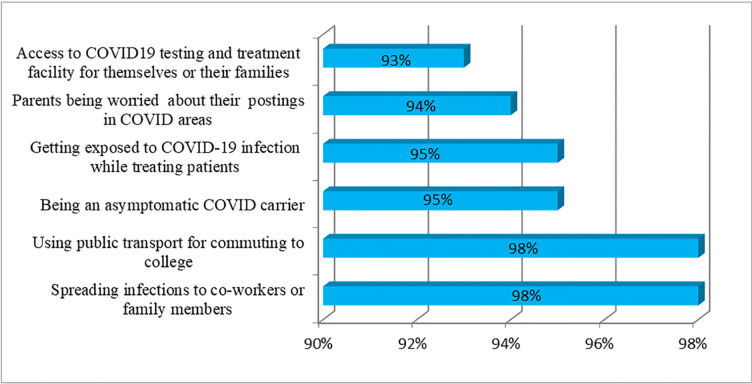
Health-related concerns* reported by the participants.*Multiple responses possible

**Figure 4:**
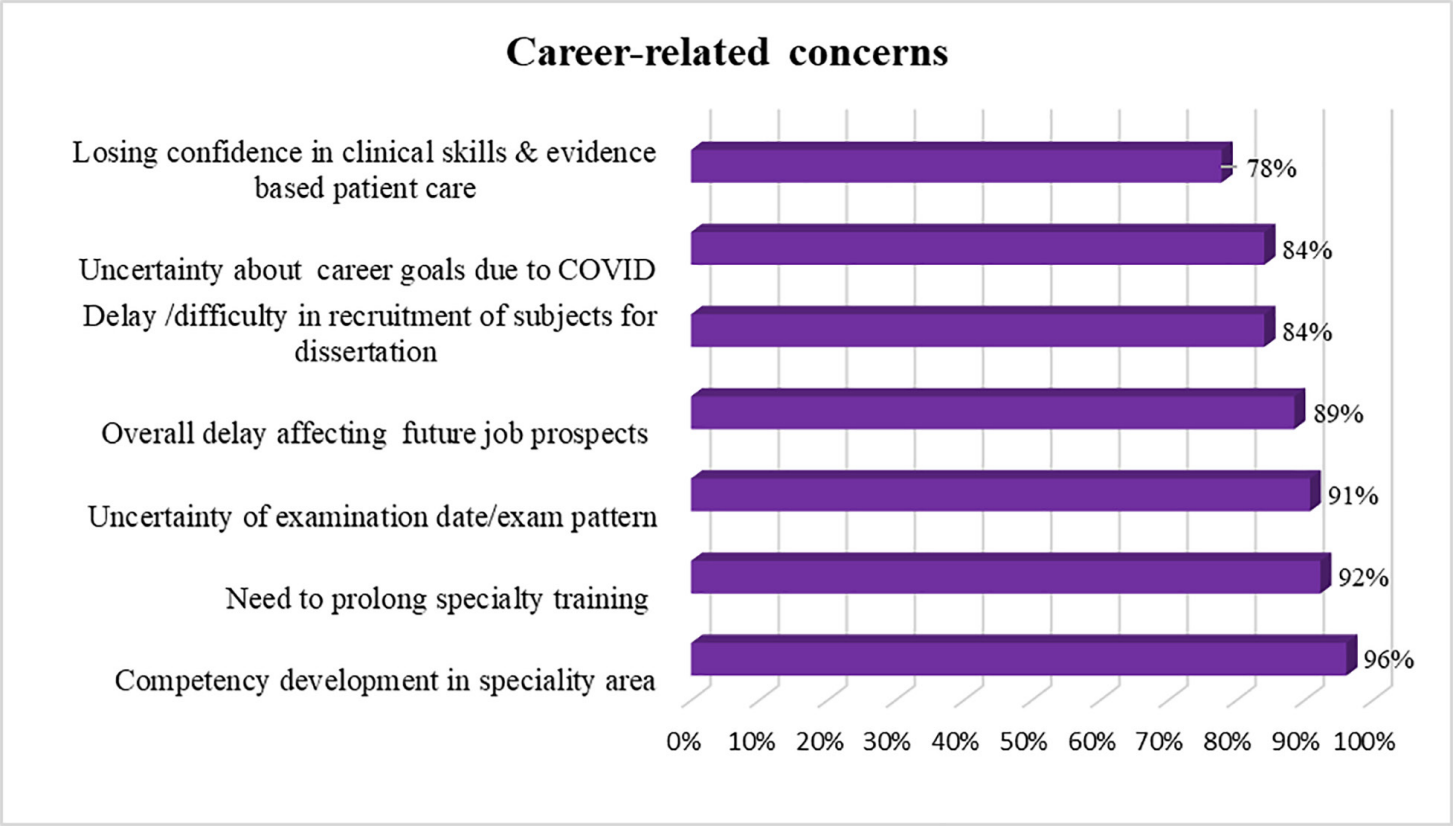
Career-related concerns* reported by the participants *Multiple responses possible

Of the total, 16% (n=21) showed COVID-19 related symptoms in the past 6 months, 16 of whom underwent COVID testing. 43% (n=9) claimed that the institution took care of their testing/treatment. 30% (n=39) of the participants had isolated themselves due to a suspected or confirmed case in their house. Figures 3 and 4 show the health and career-related concerns raised by the participants.

## Discussion

To the best of our knowledge, this is the first study to extensively evaluate the impact of COVID-19 on PG Physiotherapy training program. Survey responses from 131 participants across the state of Maharashtra, India, indicated that COVID-19 has had a profound adverse effect on not only teaching and learning, but also their psychological well-being. 

The key findings observed in this survey was that PG students felt that the pandemic had affected their clinical exposure in their PG specialty areas; the adopted online/virtual teaching method did not help much in achieving their learning objectives; also, uncertainty of examinations, delay in research related tasks and fear of contracting COVID infection were additional burdens. In response to the lockdown, various institutions and hospitals, in an effort to mitigate the spread of infection, cancelled the academic training and teaching schedule immediately, ( 4, 8, 15) causing severe disruption of the overall learning of the students not only in India, but also worldwide. However, it was observed that measures to maintain a structured learning was undertaken through different digital platforms such as Zoom, Webex, and Google meets. This led to trainees spending more time in attending online webinars, CMEs, social media surfing, with less time dedicated to clinical duties during this period. Though virtual learning methods are known to be a safer option to limit the chances of exposure to the virus along with ensuring non-disruption of teaching activities, it needs advanced infrastructural facility and limits interaction between the faculty and trainees ( 16). Iyengar et al. ( 17) quoted that COVID-19 pandemic had been a learning opportunity which appeared to have been a catalyst for new, innovative ways of delivering teaching and training. As reported in this survey, participants were involved in various newer tasks such as preparing audios/videos for patient treatment and UG training and preparing handbooks/information booklets for COVID patients. Additionally, they reported to have spent more dedicated hours in PG activities like journal club sessions, as compared to pre-COVID times. These possibly should have helped them achieve some of their missed learning opportunities. Surprisingly, despite these student engagement activities, a vast majority of participants in this study felt that they had not gained the expected hands-on skills and were unsuccessful in achieving the desired learning outcomes. Lack of learning achieved through interaction with their faculty members, peers and seniors could have possibly been a major deterrent to their learning experience.

PG physiotherapy programs in India are of a fixed tenure (2 years). It necessitates clinical hands-on practice in hospitals, hours of self-studying, reviewing literature for research, journal club presentations, and microteaching sessions for undergraduates. The pandemic has resulted in the loss of precious time of approximately 2-3 months from training activities. Besides, trainees, irrespective of their PG specialty, were expected to offer clinical services in COVID ICUs and wards which was not a part of their routine specialty clinical posting. This paradigm shift in care away from specialty training led to loss of pivotal learning opportunities needed to gain the expected competencies. As a consequence, it could be anticipated that the current PG trainees may lack proficiency in essential hands-on skills. This is in the same line with our survey where the majority of participants did not feel competent enough to work confidently after PG completion in their specialty area ( 18).

Participants in our study specifically reported that their basic assessment and management skills as well as their overall treatment approach had been altered probably due to incorporation of minimal contact practice. Cancellation and postponement of elective surgeries, reduction in outpatient and in-patient volume could have also taken a significant toll on PT referrals across all specialties, thereby limiting their clinical education. A large number of institutions adopted policies in the form of rotational postings in COVID and non-COVID clinical areas ( 12). Evidence of diminished training opportunities and disruption of medical education during the current pandemic as well as historically has been well documented ( 19). This gave way to emergence of compensatory interventions in the form of tele-rehabilitation and dissemination of therapy using videos and information booklets, thus helping students to maintain some levels of structured learning ( 20, 21). However, these compensatory techniques do not involve traditional physical examination and hands-on therapy which forms the fundamental basis of PT practice ( 18). It is worth mentioning that although the participants were involved in COVID services, very few were actively involved in any COVID-related research. We believe that involving PG students in any form of research could definitely help them develop evidence-based approach and clinical reasoning skills, thereby taking care of loss of training that was perceived by them.

As the current post-graduates are going to be future professionals, their self-perceived incompetency and their lack of confidence in following evidence-based practice could be a major hurdle in their future job prospects. This could have long term implications on the health care needs of the whole society. Therefore, finding a new balance between service provision and training becomes the need of the hour to avoid further resentment among the trainees. Also, as reiterated by Caruana et al., students can be sensitized to the fact that every clinical encounter must be embraced as an invaluable learning opportunity in times of reduced caseload ( 12).

COVID-19 pandemic caused a worldwide cancellation or postponement of examinations across all professional fields. Lack of communication from the regulatory bodies could have led to uncertainty and anxiety amongst PG trainees appearing for exit exams. The impact of this was seen in this survey wherein a large number of students reported concerns about the examination dates and pattern, lack of confidence and preparedness in appearing for the upcoming exam. This ambiguity might have resulted in the participants being unsure about their career goals which possibly could impact their future job opportunities. Similar findings were supported by Upadhyaya et al. ( 11) in their study on postgraduate orthopedic trainees in New Delhi.

This pandemic has coerced the PG students in an intractable situation in their research-related tasks as well ( 9- 12, 15, 16). Numerous factors such as reduced patient load owing to the cancellation of surgeries and referrals, decreased patient inflow due to fear of contracting infections, type of study, restrictions in the use of specialized equipment and delays in approval from universities have created major hurdles for participants in carrying out their research task ( 11). Our data corroborate with the above reasons. This might have prompted a considerable proportion of participants to suggest measures to alleviate these factors through relaxation in sample size, duration of the study intervention, and provision for change in the study design.

It was noted in this survey that though a large number of participants were involved in COVID services, a very few developed COVID symptoms in the past 6 months. Provision of timely PPE training, bathing facilities during COVID postings, and post-COVID leaves, as reported in this survey, could have taken care of their safety as well as apprehension. Post-graduate students and trainees were exposed to stressful life owing to multiple roles/responsibilities as a part of their program. COVID-19 pandemic could have additionally magnified their mental unrest, thus impacting their mental well-being ( 22). This survey has highlighted several aspects of their concerns related to their health and professional goals. Students were not only scared of getting self-exposed to COVID-19, but also worried that their family might get infected. In this survey, many reported that their family members were more anxious about them being a part of COVID workforce, adding on to their stress. Using public transport for commuting to the hospital was another major cause for distress. Hence, it is advisable for the institutions to provide counseling services and support strategies to proactively address anxiety that is required for the sound mental well-being of the students ( 9, 11).

As countries are engaged in collaborative endeavors to tackle the global impact of COVID-19 pandemic, we hope that our findings will help shape future strategies to alleviate the blow on Physiotherapy PG program. This work emphasizes several important aspects of PG trainees at such an overwhelming time and provides us with a blue-print of the early impact of the pandemic on PG Physiotherapy trainees in India. 

### Strengths of the study

This was the first study undertaken to evaluate the impact of COVID on all aspects of a Physiotherapy PG training ranging from academic, clinical, research to psychological domains during the time when pandemic effect was at its peak. The findings of this study would certainly aid in extraction of information that could help the stakeholders to devise corrective/preventive measures during such crucial times. The questionnaire, though extensive, underwent rigorous revisions by a consensus panelist to ensure robustness of the survey tool. The survey was open for a very short period of time to ensure that the dynamic nature of pandemic and the changing government/institution policies do not alter/affect the study results. Researchers tried to target the participants from various government and private Physiotherapy colleges within Maharashtra, thus ensuring effective representation of the sample.

### Weakness

Self-reported nature of this survey might have introduced social desirability bias which could have led the participants to give socially accepted answers. Online nature of data collection and snowball nature of sampling technique could have possibly over- or under-estimated the response rate. Length of the survey tool could have presumably affected the readiness of the participants to respond accurately.

### Limitations

The authors acknowledge the limitations encountered during this work. Since the sample was collected from a single state of Maharashtra, it could affect the generalizability of the findings to Indian population. Considering the time frame of data collection owing to uncertainty of pandemic and its implications, the researchers could not check the validity and reliability of the tool used. However, quality of the tool was ensured by subjecting it to multiple revisions by a team of expert panelist and piloting it before the survey. The data could also have been subjected to researcher bias. Additionally, authors did not calculate the proportion of participant responses from each institute separately, which could have been a probable source of variability in findings. Also, overall the pandemic had undoubtedly caused psychological stressors that could probably impact the participants’ responses. However, this study did not use any objective scale to assess psychological state of the participants that could justify these findings.

## Conclusion

This is one the surveys targeting the impact of COVID-19 pandemic and gives a general overview of the disruption caused in educational and training realm of Physiotherapy post-graduate students. It, thus, becomes the primary responsibility of governing bodies and educational institutes to urgently build up a resilient educational system to ensure that the pandemic does not leave long-lasting deleterious effect on these emerging professionals. 
